# Elaboration of the Gothenburg model of person‐centred care

**DOI:** 10.1111/hex.12468

**Published:** 2016-05-18

**Authors:** Nicky Britten, Lucy Moore, Doris Lydahl, Oncel Naldemirci, Mark Elam, Axel Wolf

**Affiliations:** ^1^ Institute of Health ResearchUniversity of Exeter Medical SchoolExeter; ^2^ Department Sociology and Work ScienceUniversity of GothenburgGothenburg; ^3^ Institute of Health and Care Sciences, Sahlgrenska Academy and Centre for Person‐Centred CareUniversity of GothenburgGothenburg

**Keywords:** documentation, narrative, partnership, person‐centred care, personhood, qualitative

## Abstract

**Background:**

Person‐centred care (PCC) is increasingly advocated as a new way of delivering health care, but there is little evidence that it is widely practised. The University of Gothenburg Centre for Person‐Centred Care (GPCC) was set up in 2010 to develop and implement person‐centred care in clinical practice on the basis of three routines. These routines are based on eliciting the patient's narrative to initiate a partnership; working the partnership to achieve commonly agreed goals; and using documentation to safeguard the partnership and record the person's narrative and shared goals.

**Objective:**

In this paper, we aimed to explore professionals' understanding of PCC routines as they implement the GPCC model in a range of different settings.

**Methods:**

We conducted a qualitative study and interviewed 18 clinician‐researchers from five health‐care professions who were working in seven diverse GPCC projects.

**Results:**

Interviewees’ accounts of PCC emphasized the ways in which persons are seen as different from patients; the variable emphasis placed on the person's goals; and the role of the person's own resources in building partnerships.

**Conclusion:**

This study illustrates what is needed for health‐care professionals to implement PCC in everyday practice: the recognition of the person is as important as the specific practical routines. Interviewees described the need to change the clinical mindset and to develop the ways of integrating people's narratives with clinical practice.

## Introduction

There is a long history of patient‐centredness within health care. Since the 1960s, there has been a growing literature on patient‐centred consultations,^1^ patient‐centredness,^2^, ^3^ shared decision making,^4^ person‐centred care 5, 6 and collaborative deliberation.^7^ A recent international review of person‐centred care identified three conceptual pillars: personhood, partnership and an overarching group of related concepts.^8^

Researchers interested in clinical practice have noted that these models of patient–professional relationships, however characterized, have not influenced the practice of health care to any appreciable extent. Internationally, Harding *et al*.^8^ concluded that the implementation of PCC in the mainstream remains tentative. Thus in 2010, with the intention of actually implementing PCC, an interdisciplinary group of clinical and non‐clinical academics set up a Swedish research centre for the study of person‐centred care in long‐term illness.^9^ The centre is the University of Gothenburg Centre for Person‐Centred Care (GPCC). Its founders proposed three ‘simple routines’ to initiate, integrate and safeguard person‐centred care in daily clinical practice, the GPCC model. The first routine serves to initiate a partnership by eliciting the patient narrative, defined as the sick person's personal account of their illness, symptoms and their impact on their life. It captures the person's suffering in the context of their everyday lifeworld, in contrast to medical narratives that reflect the process of diagnosing and treating the disease. The second routine serves to work the partnership by means of shared decision making, so that professionals, patients and very often their relatives all work together to achieve commonly agreed goals. The third routine serves to safeguard the partnership by documenting the narrative in the form of patient preferences and values, as well as involvement in care and treatment decision making. These routines represent clinical tasks to be undertaken by the professional as well everyday goals undertaken by the patient/relatives. This distinctive model of person‐centred care is intended to differentiate it from other models by focusing more on the capabilities of the person and is rooted in the philosophical literature.^10^, ^11^ The architects of GPCC pointedly avoided using the term ‘patient‐centred care’, arguing that the word ‘patient’ tends to objectify and reduce the person to a mere recipient of medical services.^9^

The centre has coordinated and partly funded about 40 studies in a wide range of clinical and community settings. All these studies aimed to implement the three routines of PCC, which their earlier and on‐going trials have shown to be effective. These trials have been conducted with people undergoing surgery for hip fracture,^12^ people hospitalized for chronic heart failure 13 and people having cardiac rehabilitation after acute coronary syndrome.^14^ The acute coronary syndrome project (see Table 1) will be referred to as the ‘index’ project because the GPCC model was developed by its authors. The routines of PCC are being constantly developed and adapted in the other GPCC projects. For PCC to become part of everyday clinical practice, health‐care professionals need to change their working practices as well as the environment within which PCC is provided.^15^ Thus, the aim of this paper was to examine the implementation of the GPCC model across a range of health‐care and community settings, starting with professionals' understandings of what it means, and thereby to elaborate their original definition of PCC.

**Table 1:** Characteristics of selected GPCC projects

Category	Acute coronary syndrome (index project)	Irritable bowel syndrome	Psychosis	Osteopathic fractures	Patient participation in hypertension treatment	Neurogenic communication disorders	Healthy ageing in migrant communities
Intervention population	People with acute CAD Symptomatic – to show effect	Men & women with IBS & no biological markers	People with psychosis	Older people with osteopathic fracture & pain Average age: 84	People over 30 years medically treated for hypertension	People with stroke or neurological disorder & staff in retirement homes	Foreign‐born older persons
Setting & speciality	Acute care & primary care Cardiology	Acute care &primary care Medicine	Four acute in‐care units Psychiatry	Acute care, community & person's home Orthopaedics	Medical outpatient clinic& primary care Medicine	Retirement homes Speech therapyLanguage rehabilitation	Community centre & person's home Occupational science Health promotion In the context of migration
Intervention purpose & outcome	To increase self‐efficacy & resumption of activities	Identify gender differences Understand illness perspectives Develop PCC dietary advice	Understand person's perspective & create a plan for social resources Reduce symptom burden, involuntary injections, hospital stay & ward burden	Reduce pain & restore function/activity through support, rehabilitation & activity prescription Reduce the length of stay in acute care‐cost efficiency	To design, develop and evaluate an interactive mobile phone‐based system to support self‐management of hypertension	Train staff in PCC communication & use staff's knowledge as a resource Emphasize the need for PCC trained staff	Promote health & normal ageing Empower participants & lift strengths through peer support Emphasize contextual perspectives
Intervention status	Completed	Planning an intervention	Planning an intervention	On‐going	Completed	Planning an intervention	On‐going
Design of research	RCT	Qualitative (group interviews & questionnaire) RCT planned	Before & after study (pre – measurement of ward culture, patient satisfaction and empowerment)	RCT	Focus groups, validation study, Before & after study of self‐reports & video recordings of consultations	Video recordings of dyads (person & HCP) used for training purposes for educating staff	RCT and implementation research

CAD, coronary artery disease; IBS, irritable bowel syndrome; PCC, person‐centred care; RCT, randomized control trial; HCP, health‐care professional.

## Methods

The research was devised and conducted by an international team based in Gothenburg, Sweden and Exeter, UK. As it was not possible to study all 40 projects, it was decided to sample seven projects that reflected primary, secondary and tertiary health‐care services (hospital, outpatient, homecare, primary care). They were chosen because they were further ahead in the implementation process. The projects were as follows: acute coronary syndrome (the index project), irritable bowel syndrome, healthy ageing in migrant communities, neurogenic communication disorders, patient participation in hypertension treatment, psychosis and osteopathic fractures (see Table 1).

Three researchers in Gothenburg conducted 17 interviews with 18 people (one interview was conducted with two people), none of whose first language was English. There were two or three interviewees from each of the selected seven projects. Interviews were conducted in either interviewees' or researchers' offices. Seven interviews were conducted by a postdoctoral researcher, eight by a PhD student and two by a professor. Six interviews were conducted in Swedish and translated into English. The interviewers used a topic guide covering the definition of PCC and its routines, and perceived differences from and similarities to previous health‐care approaches such as evidence‐based medicine and patient‐centred care. All the interviewees were health‐care professionals, and the sample consisted of ten nurses, three occupational therapists, two speech and language therapists, two physicians and one clinical psychologist. They will be referred to as R1‐R18. Reflecting the interviewees’ language, we will use both the terms ‘patient’ and ‘person’ when presenting the results. The interviews lasted for about an hour, and the range was 45–78 min.

The English language interviews were transcribed in the UK. All the transcripts were analysed first by the two UK‐based researchers, who drew up and applied a preliminary inductive coding frame. The emergent themes were as follows: descriptions of PCC; the patient's account; the philosophy; differences in thinking and seeing; the approach to working; the group; the intervention; and barriers. The coding frame was developed over time with the rest of the team and discussed in regular online and face‐to‐face meetings. A subsequent comparative framework based on interviewees’ understandings of the three routines provided the basis of a second deductive coding exercise for this paper. This framework was discussed in a face‐to‐face meeting with some of the interviewees, who made comments and corrections that were subsequently incorporated.

In this paper, we are not aiming to compare the projects with one another. Rather we are using data from seven diverse projects to explore different professionals’ understanding of the GPCC routines in a range of settings, and how they had changed their clinical practice. The results are presented to demonstrate that different interviewees emphasized different aspects of PCC. They are summarized as a series of descriptions of PCC, as articulated by interviewees, which elaborate the three routines. We will explore the effect of context on the implementation of PCC in particular settings of another paper.^16^

## Results

In a country where patient‐centredness is not well established,^17^ interviewees were not simply reiterating programmatic statements about what they should be doing, but explained their own understanding of what PCC means in practice and the challenges they encountered in trying to implement the GPCC model. One interviewee said that:

I think it's a huge process to go from usual care to person‐centred care… the change could sometimes seem very small but … it changes everything

(R8).

Interviewees drew both from their own previous professional experiences and from the experience of implementing the GPCC model. Their understandings of PCC were shaped by their practice setting and the nature of the population they were caring for. The results will be presented in four sections: the general description of PCC, the narrative, the partnership and the documentation.

### General description of PCC

When asked to define PCC, several interviewees began by explaining the difference between a patient and a person. They said that a patient can be objectified as something which can be measured, but to describe a person, one needs to go beyond objective biomedical measurements. It was explained that if you are a person, ‘you are not your disease’ (R12). People may have the same disease but not everyone is affected in the same way. A patient is a temporary role taken on in the context of health care:

there is an idea behind just calling it person‐centred instead of patient, and that is that they are mostly *not* patients

(R6).

The person's own opinions are more important than when they are seen as merely a patient; as a person, their emotions, wishes, resources, environment and community participation may all be acknowledged.

The difference between patient‐centred and person‐centred care was explored in the interviews. For one interviewee, there were no major differences. For another who felt that one term (person‐centredness) would likely replace the other, there were no conflicts between the two. However, other interviewees described clear differences between the two terms. It was asserted that patient‐centred care involved no theory around the patient, no change in attitude and no exploration of people's emotions, wishes and resources. By contrast, person‐centred care was based on a philosophy of the person; some interviewees talked about ‘centring on the person’ (R4). It was claimed that patient‐centred care was concerned with groups of people (e.g. people with diabetes), while person‐centred care focuses on individuals. In patient‐centred care, an assistive device would be provided without checking with the individual what they wanted:

…providing a wheelchair without discussing it with the person.. to me, is the opposite of person‐centredness. I always want to discuss what do you need this assistive device for?

(R16)

It was clear from some of the interviews that person‐centred care required health‐care professionals to view the people they cared for in a different way. In usual care, professionals talk above patients or about them, but patients are never part of the team. The challenge in PCC was to see patients as human beings with the same needs for love and security as anyone else. To use the word ‘person’ rather than ‘patient’ could change the professional's mindset. One interviewee said that she tried to encounter people in the way she would want to be encountered herself. The difficulty for mental health‐care professionals in particular was that they only see people when they are ill. One interviewee said

…but when you meet the person in the city, all of a sudden and see that “‘Oh God! This is *this* person, you wouldn't have thought”… that's good, because then you see that there is hope and there is actually a very big change in the person now compared to when he or she was in the ward

(R14).

In person‐centred care, the person is viewed the same way as any other person, and the challenge for the professional is to show the person that they have been seen. It requires a different approach based on flexibility and open‐mindedness. Rather than making assumptions based on previous experience with the same patient population, professionals need to see every person as a new person. In PCC, professionals need to see that, even if severely ill, people are experts on their own lives and have resources. Therefore, professionals need to acknowledge the person's life, social context, knowledge and capacities as well as their shortcomings. The point was also made that people need to be active and take some responsibility for their own health, and one interviewee described the ‘persons’ in PCC as those who co‐operate with carers to produce their own care.

Several interviewees mentioned that persons always have some resources, and a recurring theme was that of the ‘capable person’. One of the respondents from the index project said that the

basic assumption is that all people are capable, including the small child and the elderly person – well they needn't be elderly, the dementia patient is capable. People are capable of different things, but everyone is capable … if the person can't give an account of themselves, of course there are relatives who can

(R5).

For some interviewees, seeing persons as capable means that they have resources and can take responsibility for their own actions; for others, it means that persons can make a contribution and support each other in community settings; or as one mental health‐care professional put it, it means that

I show them that you are seen and you are a capable person … your dreams or your goals count and they have value

(R13).

Person‐centred care can help to ‘lift the strengths’ (R16) of individuals and, by showing the person their own potential, professionals can give them hope and thereby assist their recovery.

One interviewee mentioned inequalities and said that in person‐centred care, everyone is seen as capable and having the same rights, not only those who can scream the loudest. It was also acknowledged that the environment can create possibilities for someone's capability to be realized.

Some interviewees emphasized the difference between PCC and biomedicine, claiming that in PCC the focus is on symptoms not signs, the person is not a passive recipient of care and is not objectified. As one interviewee explained:

.. (medical care) had developed into objectifying the patient and reflecting patients as an object which you can measure.. this has been the major difference

(R7).

### Narrative

Interviewees discussed narratives in a taken‐for‐granted manner without reference to writings on narrative‐based medicine,^18^, ^19^ for example, but congruent with the view that narratives provide insights about individuals rather than so‐called typical patients. Patients' narratives were generated in several different ways in the different projects; mostly, they were generated within the consultation, but in some cases narratives were generated through the use of technology. Several interviewees emphasized the importance of listening to the person, and one made the distinction between listening and hearing. For this professional, listening was much stronger than hearing. One professional said:

I feel I have got it right when … they speak and they talk about their troubles and, er, I shut up! (laughs)

(R6).

Interviewees gave examples of the kinds of open questions that could be used to generate the person's narrative. These included questions about the person's feelings about the current situation; asking people what they wanted to discuss; enquiring about thoughts and beliefs as well as symptoms. One interviewee, talking about the index project, said that there were three open questions required to obtain the person's narrative. The first is to ask why the person sought treatment; the second is to find out the person's goal; and the third is to find out how much the person is prepared to do to achieve the goal, in other words their motivation and resources. For the individual concerned, it is a chance to tell their story. Some people may be able to develop insights into their own difficulties by telling their stories. For people with neurogenic communication disorders, it may be necessary to use other methods such as pictures, photographs, body language or talking to family members; these may generate non‐verbal ‘narratives’. In the hypertension project, narratives were generated by individuals recording data on their own mobile phones.

In some projects, the person's own goals were seen as central to the narrative, while in others they were not explicitly acknowledged or even mentioned. One interviewee said that

.. in person‐centred care the goal is what the person says is the goal and then the surgical procedure is just a tool, a means to achieve the goal

(R8).

Other formulations of this view were that professionals needed to focus on the person's goals and not the professional's goals, or that the person's goals formed the starting point of care. Interviewees gave a wide variety of examples of people's goals, all of which were based on the lifeworld rather than reflecting biomedical goals. These included picking mushrooms in the forest; digging a potato patch; walking the dog, as well as common goals such as having a job or a partner. One interviewee working with older people from immigrant backgrounds said:

Maybe it's not so important to be able to go to the toilet independently, it might be more important to focus on, “I want to go to the book club once a week”

(R16).

By engaging with the person's lifeworld goals, it may be easier to discuss the professional's goals such as giving up smoking.

Interviewees also gave examples of situations in which they tried to modify the person's goals by setting interim goals or changing unrealistic goals. Some pointed out the need to help patients formulate interim goals if their goals were not easily reached. Interviewees also said they helped people to think about what resources might be needed for them to achieve their goals. One interviewee talked about developing their intervention around the activity goals of each person, to help them achieve what they wanted to achieve. For most interviewees, establishing the person's goals could take time.

Some interviewees talked about problematic aspects of people's goals and how they managed in such cases. If the person's goal conflicted with the professional's goal, interviewees had different responses depending on the context. In some cases, they simply respected the person's opinion. In other cases, professionals could struggle if the patient wanted to do something different from what they had advised or recommended. Interviewees said that if patients' goals were unrealistic, they could sometimes be broken down into smaller steps, or they might try to shift the person's goals to more realistic ones:

..the patient was asked “What are your goals?” and he said “To be able to drink as much alcohol as I can get hold of” …. But what our very … experienced doctor who asked that question and got that answer, she said “Well, maybe we could think about other things that give your life meaning”

(R15).

Professionals could not support the goals of people suffering from paranoia, or dangerous goals such as the wish to keep a weapon at home. Some interviewees spoke about how health‐care professionals could skilfully change someone's perception and therefore their goals.

Thus, interviewees saw the purposes of the narrative as a way of showing patients that they are seen as people; establishing their goals; understanding the context of treatment or why people may not be interested in professional advice; and as the basis for agreement and partnership.

### Partnership

The partnership between persons and professionals was described differently by various interviewees, depending on the nature of the patient population and the context of the project. While the routine of narrative involved the elicitation of goals, for several interviewees partnership was about reaching agreement about shared goals. To reach the agreement, professionals may have to identify the person's resources and their need for support, to help them achieve their goals. For one professional, ‘success is the actual agreement’ (R5). Some interviewees talked about the person as an active partner. In the context of rehabilitation, one commented that it is impossible to do rehabilitation if the person is not involved. To make the person an active partner, the professional may need to use the person's capacity and resources in addition to their own knowledge, skills and understanding. One interviewee described PCC as:

… listening and identifying and agreeing with the patient on what resources they have and what support they need from us to reach that goal

(R5).

It was recognized that this places demands on individuals because they have to do most of the work and take responsibility; it was also recognized that not everyone wants, or is able, to do this. Some interviewees talked about patients as equal partners, because patients are expert in themselves and in their experiences of illness. This meant that conversations were different and the rules were changed. As an equal discussion partner, the patient can choose what to discuss. In the hypertension project, possession of one's own data could change the relationship between patient and professional. Clearly, communication is central to this kind of partnership. However, several interviewees noted constraints on the partnership conceived in this way. As one said

even if you're person‐centred you can't be someone's best friend because you're still there to do your job in the context of health care

(R18).

If the person is suffering from psychotic symptoms, they may need to take medication before it is possible to ‘share worlds’ (R14). Some respondents commented on the tension between doing what the person wants and being professional; others noted that they acknowledged the limits of what they could do, sometimes referring patients to other agencies or networks. Several respondents talked about the person's social network, either through the inclusion of family and friends in consultations, or more formally through the establishment of a social resource group as in the psychosis project. In this partnership, the role of the health‐care professional may be transformed from a provider of care to a consultant, adviser, administrator or facilitator. One explained:

It's more like a discussion partner who can talk about what's evidence based and what could be good for you and are there any difficulties in your life that you cannot solve at the moment

(R16).

Some professionals commented on the need to be self‐critical in this process, to be aware of their own approach and to ensure that they had really listened.

### Documentation

In the original characterization of PCC, documentation was seen as a way of safeguarding the partnership and recording the person's narrative. Ekman *et al*.^9^ argued that this documentation gives legitimacy to patient perspectives, makes the patient–provider interplay transparent and facilitates continuity of care.

**Figure 1:**
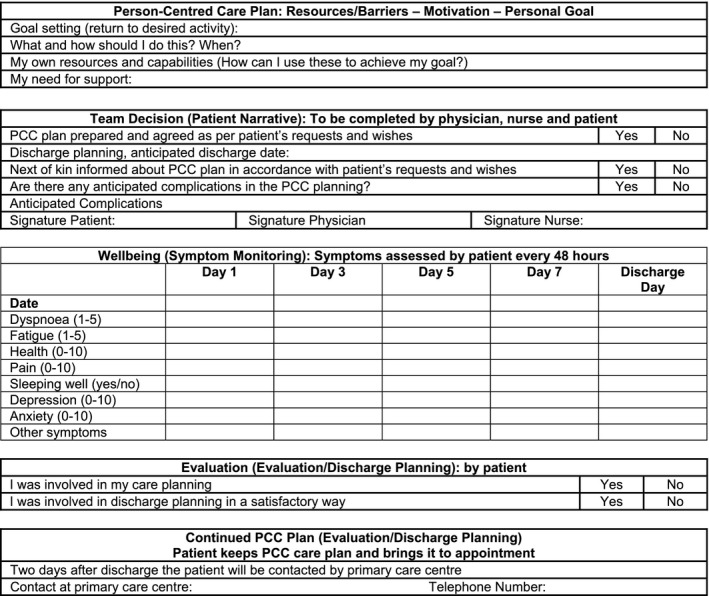
Example of person‐centred health plan.

Unsurprisingly, the index project had the most well‐developed procedures for documentation. The person‐centred health plan (see Fig. 1) contains a symptom diary which people complete every second day while in hospital; this is jointly discussed and re‐evaluated on a regular basis during the medical rounds. The person receives a copy of the PCC plan, and at discharge, it is discussed with the responsible primary care health‐care provider. During each visit at the outpatient clinic or primary care clinic, the PCC plan should be discussed, evaluated and, if necessary, revised together with the person. The PCC health plan is therefore seen as a way of ‘guaranteeing the care chain’, in other words making sure that information about each person is shared with other health‐care professionals caring for that person. Individuals are given all the documentation when they leave hospital, including test results, discharge summaries and the PCC health plan itself. The health plan can also be used to ensure that the person really did receive person‐centred care.

Interviewees in other projects talked about documentation in two ways: as part of clinical practice and as a resource for individuals. They said that some of the other projects have clear documentation as part of their clinical practice or their intervention and some do not. Some interviewees described challenges to integrating PCC with medical records. One said that even if one professional makes a record in the patient's chart, it is not always transferred to other staff. Even though the patient's narrative was prioritized, the documentation system did not always have enough space or the right structure for recording these narratives:

.. there's all kinds of subheadings… it just makes it, fragments it, you have to write one thing here, there's no way to document a narrative

(R6).

Another said that ‘documentation is a huge problem….that so far we have always used paper’ (R8) but this caused difficulty in transferring their own system of documentation to an electronic record.

As a resource for individuals, some projects had developed documents appropriate to their own settings including a health diary, journal writing and notebooks as well as a website containing a health diary option, chat function and the technology for people to monitor their own health status. One interviewee talked about

…notebooks that (we) give to the patients from the beginning…also space where they can write their own thoughts…what kind of questions they want to ask the doctor

(R13).

A range of technologies are used for documentation, both paper based and electronic. In a couple of projects, people were able to use mobile phone technology to record data about themselves. Interviewees said that this could increase patients' understanding of their own functioning and to help them make connections between functioning and health status.

Some forms of documentation include data provided directly by the individual, while others are administered by professionals. As in the index project, documentation could provide a means of sharing information with the person, or developing jointly written care plans. Talking of the latter, one interviewee said:

..the care plan needs to be documented very.. meticulously..otherwise you can't really tell at all whether the patient has been given person‐centred care or not..we need to figure out, then, in the group…what should they write as documentation

(R14).

## Conclusions

The problem which the architects of GPCC aimed to address is the paucity of person‐centred practitioners.^20^ In this paper, we have shown how professionals who are implementing an evidence‐based model of PCC have translated it into their own settings. In the process of turning theory into practice, they have elaborated the programmatic statements in original GPCC model of person‐centred care. The model has been evaluated using randomized controlled trials with people experiencing hip fracture, heart failure and acute coronary syndrome.^12^, ^13^, ^14^ In the present study, the three routines were implemented in several other settings, despite some barriers and tensions.^16^, ^21^

Reeve has pointed out that there is no definition of the person in patient‐centred care,^22^ and this study shows that articulation of the person was at least as important as the three practical routines of PCC. In the words of one interviewee:

the main problem with implementing person‐centred care is that you can't tell anyone how to approach people, it's not about what you do it's more about how you are

(R16).

For several interviewees, a central feature of PCC was the difference between a person and a patient, and the importance of seeing the ‘patient’ differently. The patient is seen as a person who is capable and resourceful despite their health problems, and whose care should be shaped by their own experiences and goals. Individuals’ goals are often absent from clinical practice 23 and are not usually measured in clinical trials and so are also absent from the evidence base.^24^

These GPCC clinician‐researchers have used frameworks from their own professional training and other sources to elaborate the original model of person‐centred care and implement it in their own settings. Seeing the patient as a person who is more than the sum of their biological parts, and as a capable stakeholder, requires a change in the mindset of clinicians, particularly those who believe they already practise in a patient‐centred way.^25^ All the interviewees perceived PCC to be different from usual care, and some explicitly said that it differed from biomedicine.

The paradox of PCC is that it focuses on persons who are initially identified and defined by their medical categories, and in this study, all the projects except one have labels based on diagnoses or risk factors. This paradox may attenuate in situations of multimorbidity, where people have been given several diagnostic labels. The GPCC model is based on routines but this study shows that the changed perceptions of professionals are at least as important as the routines; all the interviewees in our study had signed up to it, but others might be less willing to do so, especially if they are unable to see the difference. The trials that have provided the evidence base for the GPCC model have used various outcome measures including length of hospital stay, functional performance, risk of readmission and quality of life.^12^, ^13^, ^14^ There may be other, as yet untested, outcome measures of greater importance to patients.

For professionals, PCC has a built‐in tension between being an advocate of the patient and supporting their goals; a professional who is governed by rules, regulations and evidence‐based guidelines; and more recently a provider of a service located within the economic marketplace. For organizations, there are several competing professional or institutional logics within the health‐care setting.^26^ It has been shown that there may be a clash between the logic of medicine (based on health outcomes) and the economic managerial logic, based on efficiency.^27^, ^28^ PCC introduces the further dimension of health as a resource for everyday life.^29^ A particular area where this clash between different logics is evident is the integration of PCC into clinical practice. Medical records are constructed within a biomedical frame designed to transform personal narratives into black and white clinically defined categories; there is no room for the colourful and idiosyncratic nature of individuals’ experiences.

Sociologists and others have argued that the emphasis on self‐management and patients' resources is part of the move to a more efficient and less costly health service, in which patients take on roles and responsibilities previously given to professionals.^30^, ^31^, ^32^ In this study, the discussion of capability was based on a philosophy of the person,^11^ rather than as part of a neoliberal strategy to shift responsibility. The engagement with people's goals was much broader than improved self‐management. One interviewee referred to cost‐effectiveness as ‘very boring words’ (R18) and others made little reference to the costs of health care.

This paper has some limitations. Most of the projects were still at the stage of developing their interventions, and so interviewees were drawing upon their experiences of the piloting and development phases of their projects as well as their previous professional experiences. As the focus is on the views of clinician‐researchers who are working to implement the GPCC routines, no service users were interviewed, but we plan to do so in the next phase of this study. Despite these limitations, the strength of the paper is in its focus on the implementation of an evidence‐based model of PCC in a wide range of clinical and non‐clinical settings. The experience of these GPCC clinician‐researchers demonstrates what is needed for health‐care professionals to become practitioners of PCC. There is a need for a changed clinical mindset, in which patients are seen and understood as persons, which is at least as important as the practical routines. There are epistemological and practical challenges in reconciling people's narratives and lifeworld goals with everyday practice. At GPCC, these challenges are being addressed through training programmes for professionals, innovative methods to make PCC routines habitual and innovative methods of documentation.

## Conflicts of interest

The authors have no conflict of interests to declare.

## References

[1] Balint M . The Doctor, His Patient and the Illness. London: Pitman, 1964. 10.1016/s0140-6736(55)91061-8 14354967

[2] Stewart M , Belle BJ , Weston WW , McWhinney IR , McWillian CL , Freeman TR . Patient‐Centred Medicine: Transforming the Clinical Method, 2nd edn Abingdon: Radcliffe Medical Press Ltd., 2003.

[3] Mead N , Bower P . Patient‐centredness: a conceptual framework and review of the empirical literature. Social Science and Medicine, 2000; 51: 1087–1110. 11005395 10.1016/s0277-9536(00)00098-8

[4] Charles C , Gafni A , Whelan T . Shared decision‐making in the medical encounter: what does it mean? (or it takes at least two to tango). Social Science and Medicine, 1997; 44: 681–692. 9032835 10.1016/s0277-9536(96)00221-3

[5] Leplege A , Gzil F , Cammelli M , Lefeve C , Pachoud B , Ville I . Person‐centredness: conceptual and historical perspectives. Disability & Rehabilitation, 2007; 29: 1555–1565. 17922326 10.1080/09638280701618661

[6] McCormack B , Karlsson B , Dewing J , Lerdal A . Exploring person‐centredness: a qualitative meta‐synthesis of four studies. Scandinavian Journal of Caring Sciences, 2010; 24: 620–634. 21050249 10.1111/j.1471-6712.2010.00814.x

[7] Elwyn G , Lloyd A , May C , van der Weijden T , Stiggelbout A , Edwards A , *et al* Collaborative deliberation: a model for patient care. Patient Education and Counseling, 2014; 97: 158–164. 25175366 10.1016/j.pec.2014.07.027

[8] Harding E , Wait S , Scrutton J . The State of Play in Person‐centred Care: A Pragmatic Review of How Person‐Centred Care is Defined, Applied and Measured. London: Health Foundation, 2015.

[9] Ekman I , Swedberg K , Taft C , Lindseth A , Norberg A , Brink E , *et al* Person‐centred care – ready for prime time. European Journal of Cardiovascular Nursing, 2011; 10: 248–251. 21764386 10.1016/j.ejcnurse.2011.06.008

[10] Ekman I , Hedman H , Swedberg K , Wallengren C . Commentary: Swedish initiative on person centred care. British Medical Journal, 2015; 350: h160. 25670185 10.1136/bmj.h160

[11] Ricoeur P . Oneself as Another. Chicago: University of Chicago Press, 1992.

[12] Olsson L‐E , Karlsson J , Ekman I . The integrated care pathway reduced the number of hospital days by half: a prospective comparative study of patients with acute hip fracture. Journal of Orthopaedic Surgery and Research, 2006; 1: 3. 17150123 10.1186/1749-799X-1-3 PMC1634996

[13] Ekman I , Wolf A , Olsson LE *et al* Effects of person‐centred care in patients with chronic heart failure: the PCC‐HF study. European Heart Journal, 2012; 33: 1112–1119. 21926072 10.1093/eurheartj/ehr306 PMC3751966

[14] Fors A , Ekman I , Taft C *et al* Person‐centred care after acute coronary syndrome, from hospital to primary care ‐ A randomised controlled trial”. International Journal of Cardiology, 2015; 187: 693–699. 25919754 10.1016/j.ijcard.2015.03.336

[15] Wolf A , Ekman I , Dellenborg L . Everyday practices at the medical ward: a 16‐month ethnographic field study. BMC Health Services Research, 2012; 12: 184. 22748059 10.1186/1472-6963-12-184 PMC3409076

[16] Moore L , Britten N , Elam M , Lydahl D , Naldemirci O , Wolf A . Implementation of person‐centred care in different contexts: identifying barriers and facilitators in practice. Scandinavian Journal of Caring Sciences, under review. 10.1111/scs.12376 PMC5724704 27859459

[17] Docteur E , Coulter A . Patient‐Centredness in Sweden's Health System – An External Assessment and Six Steps for Progress. Stockholm: Vardanalys, 2012.

[18] Greenhalgh T , Hurwitz B . Narrative Based Medicine: Dialogue and Discourse in Clinical Practice. London: BMJ Books, 1998.

[19] Charon R . Narrative Medicine: Honouring the Stories of Illness. New York: Oxford University Press, 2006.

[20] Hawkes N . Seeing things from the patients' view: what will it take? British Medical Journal, 2015; 350: g7757. 25670180 10.1136/bmj.g7757

[21] Naldemirci O , Lydahl D , Britten N , Elam M , Moore L , Wolf A . Tenacious assumptions: exploring the tensions and variations in person‐centred care. Health, under review. 10.1177/1363459316677627 27879342

[22] Reeve J . Interpretive medicine: supporting generalism in a changing primary care world. London: Royal College of General Practitioners. Occasional Paper, 2010; 88. PMC3259801 21805819

[23] Denford S , Frost J , Dieppe P , Cooper C , Britten N . Individualisation of drug treatments for patients with long‐term conditions: a review of concepts. BMJ Open, 2014; 4: e004172. 10.1136/bmjopen-2013-004172 PMC3975745 24670429

[24] Wicks P . Measuring what matters: the case for patient generated PROMS. British Medical Journal, 2015; 350: h54. 25670198 10.1136/bmj.h54

[25] Eaton S , Roberts S , Turner B . Delivering person‐centred care in long term conditions. British Medical Journal, 2015; 350: h181. 25670186 10.1136/bmj.h181

[26] van den Broek J , Boselie P , Paauwe J . Multiple institutional logics in health care: ‘productive ward: releasing time to care’. Public Management Review, 2014; 16: 1–20.

[27] Wikström E , Dellve L . Contemporary leadership in healthcare organizations. Journal of Health Organization and Management, 2009; 23: 411–428. 19862865 10.1108/14777260910979308

[28] Lindberg K . Performing multiple logics in practice. Scandinavian Journal of Management, 2014; 30: 485–497.

[29] Reeve J , Blakeman T , Freeman GK *et al* Generalist solutions to complex problems: generating practice‐based evidence – the example of managing multi‐morbidity. BMC Family Practice, 2013; 14: 112. 23919296 10.1186/1471-2296-14-112 PMC3750615

[30] Mol A . What diagnostic devices do: the case of blood sugar measurement. Theoretical Medicine and Bioethics, 2000; 21: 9–22. 10927966 10.1023/a:1009999119586

[31] Rogers A , Kennedy A , Nelson E , Robinson A . Uncovering the limits of patient centeredness: implementing a self‐management trial for chronic illness. Qualitative Health Research, 2005; 15: 224–239. 15611205 10.1177/1049732304272048

[32] Taylor D , Bury M . Chronic illness, expert patients and care transition. Sociology of Health and Illness, 2007; 29: 27–45. 17286704 10.1111/j.1467-9566.2007.00516.x

